# Expanding Access to Social Support in Primary Care *via* Telemedicine: A Pilot Study

**DOI:** 10.3389/fpsyt.2022.795296

**Published:** 2022-02-03

**Authors:** David C. Fipps, Kristin S. Vickers, Beth Bergstedt, Mark D. Williams

**Affiliations:** ^1^Department of Psychiatry and Psychology, Mayo Clinic, Rochester, MN, United States; ^2^Social Work, Mayo Clinic, Rochester, MN, United States

**Keywords:** pilot study, social determinants of health, coronavirus, COVID-19, telehealth, social work, primary care

## Abstract

The coronavirus pandemic quickly exposed the need for efficient and widespread implementation of telehealth services. Additionally, it further unveiled the impact of social and environmental barriers to healthcare in underserved, rural populations. This in-practice pilot study tested the utility of a geographically centralized social worker providing services between a patient and a primary care provider via telecommunication at two high volume rural outpatient family practice clinics. Outcome measures included patient and provider satisfaction. Twenty-two telehealth social work encounters occurred spanning both adult and pediatric patients. Data collected from patients, primary care providers, and social work staff revealed positive feedback. The data from our small pilot study demonstrated that social work triage delivered via a tablet was an acceptable and valued resource in busy primary care practices.

## Introduction

The coronavirus pandemic has opened multiple avenues of rapid advancements in methods of healthcare delivery with telemedicine being at the forefront of this change. Many clinical settings required rapid adjustments to their abilities to deliver services to patients safely. However, this pandemic also acted as a catalyst to further unveil the impact of social and environmental barriers to healthcare on underserved populations (1). These barriers, termed social determinants of health (SDOH), hold a strong influence on the health outcomes seen by many populations (2). These factors have been defined in various ways, but often include both environmental factors (e.g., transportation), and patient factors (depression). Primary care clinics have longitudinal relationships with their patients, allowing for more chances of identifying and addressing these SDOH (3). However, as primary care physicians (PCP) are generally tasked with an already heavy clinical patient load, they may feel they lack both the time and expertise, to address these social determinants of health (4). Social workers (SW) are often used as a conduit to address these contexts; however, unfortunately, there is a significant shortage of social work staff.

It has been estimated that 1 in 5 counties in the US have at least some unmet need for non-prescribing mental health professionals, and 8% of US counties have a severe shortage with over half of their needs unmet (5). In this study, rurality and per capita income were the best predictors of unmet need with a 1-point increase in rurality (on a 9-pouint Rural-Urban Continuum Code) corresponding to an increase in unmet need of 3.3 percentage points (5). Estimates from the National Association of Social workers indicate that more than 80 percent of licensed social workers who provide services to older adults practice in metropolitan areas, whereas only 3% practice in rural areas (6).

Primary care social work interventions have shown positive outcomes in reducing higher cost utilization (7, 8), improving mental health symptoms (9–11), improved medication and diet adherence (11), and improved social functioning (10). However, differentiation between on-site and virtual social work services is under studied. In some of the above literature, part of the intervention (especially follow up case management) occurred by phone. A systematic review of 10 years of technology-based interventions in social work practice (12) found only 6 small studies (out of 87 screened) describing a variety of approaches and targets. None of these were in primary care settings and the potential benefit of extended reach was described along with many of the challenges inherent in using technology (patient technical comfort and knowledge, concerns about confidentiality and privacy, and provider challenges with feasibility). Positive outcomes were in the categories of anxiety reduction, improved well-being, and staff satisfaction.

Delivering virtual SW services is not a new concept, however there is very little data regarding acceptability and best practices. As the coronavirus pandemic has catalyzed the use of virtual technology for patient care, there is clearly a need for data on evidence-based approaches to virtual encounters. In this context, this in-practice pilot study sought to explore the acceptability of a social work contact aimed at identifying and addressing emotional and social challenges (SDOH) delivered in real time via an electronic device from a central location. This study tested the utility of this immediate in-visit social work access delivered to primary care patients in two high-volume outpatient clinics. As there is currently a dearth of published information on the practicality of having a SW virtually imbedded as a part of the primary care team, this pilot study aimed to evaluate the acceptability of a geographically centralized SW providing services at the point of contact between a patient and PCP via the use of telecommunication technology. Furthermore, this study aimed to determine the best practice methods to efficiently accomplish this task with outcome measures focused on the satisfaction of both patients and healthcare staff.

## Methods

This mixed method study as part of an in-practice pilot was presented to the Mayo Clinic Institutional Review Board (IRB) and was determined to not require formal review by the board. The study setting was two family medicine sites in Rochester, MN, a city of over 120,000 and the third largest city in Minnesota. The study started at one clinic (30 PCPs with a panel size of 23,996 patients) and then expanded to include an additional clinic (22 PCPs with a panel size of 18,926 patients). While Mayo Clinic primary care clinics (91 in total) are spread in much more rural settings, two Rochester sites were chosen as pilot sites due to rural clinic internet bandwidth concerns at that time. A team was organized that included telemedicine experts, social work and mental health providers, primary care and desk representatives, a qualitative researcher, and appropriate administrative staff. The research team consulted the telemedicine experts on how to appropriately utilize tele communication technology for this specific aims of this project. The identified goal was to find a tool that would allow a distant social worker to interact in real time visually and verbally with a patient. This led to review of various electronic hardware and an electronic tablet was chosen based on several advantages including ease of use, mobility, the capability to control access to the institutions' intranet, and good visibility to the patient of a virtual social worker. A tablet was able to be available in any exam room that would be something easy for patients and providers to initiate.

### Acceptability

**Figure 1:**
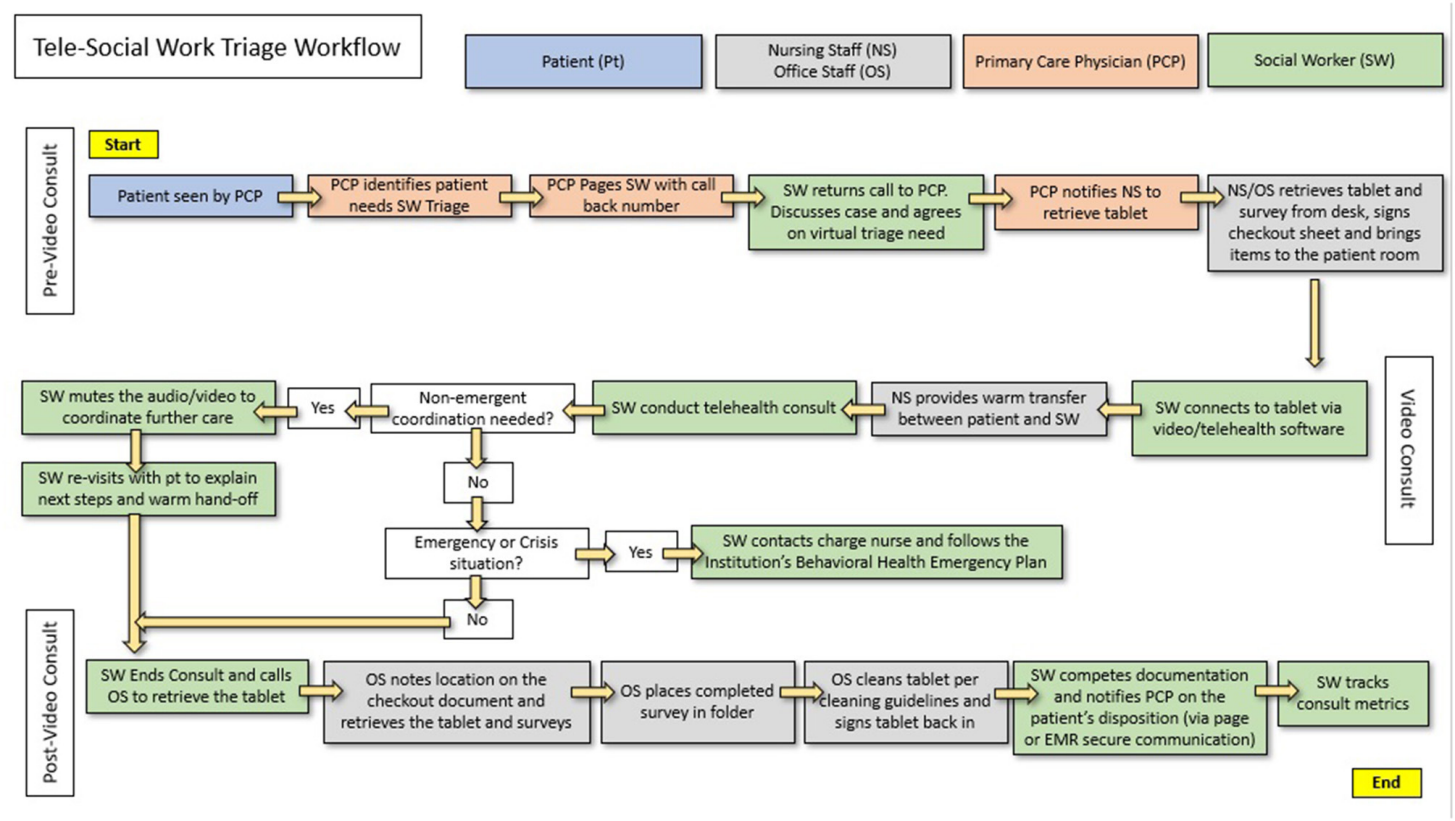
Tele-social work triage workflow starting with the patient being seen by the primary care physician, followed by the tele-social work encounter, ending in consolidation and tracking of the consultation metrics.

Using quality improvement plan-do-study-act (PDSA) cycles as described in the literature (13) this study initially explored issues that could arise around the location of the tablet within the clinic, how to ensure it was quickly brought to the room where a primary provider was seeing a patient and then cleaned and returned for the next patient, ways to notify the social worker when the patient was ready, and what to do in an emergency situation. From this acceptability workflow, the social worker collected data on all requests, her ability to respond, and estimates of resources needed (see Figure 1). The study initially started in one primary care physician clinic, but then spread to a 2nd clinic. Requests for social work point-of-care input (described as triage visits), missed opportunities, and patient and provider satisfaction with virtual triage were tracked throughout the study. Data were captured by on-site and Licensed Clinical Social Workers along with PDSA cycles on barriers, surveys, and interviews for patients and providers on satisfaction.

### Patient and Physician Satisfaction

The study targeted 30–50 triage visits to develop confidence in process and patient and provider opinion. Upon identifying low demand, missed opportunities, or negative feedback from patients or providers, the research team explored root causes. PDSA cycles of change were used to test ways to increase effective use of the service offered.

### Key Informants/Participants

To determine acceptability of these virtual social work consultations in primary care, three key informants were identified (1) the primary care providers ordering the social work consultation; (2) the patients participating in virtual social work consultation; and (3) social workers delivering the virtual consultation. Because this pilot was occurring during normal operation of the clinic and with actual patient visits, the design aimed to be minimally disruptive to patients, providers, social worker, and clinic staff. Data collection methods were designed to capture brief real-time feedback about the implementation of virtual social work visits.

### Data Collection

#### Patient Opinion Data

A brief 3-item survey was developed to assess patient experience with virtual social work visit immediately following the visit. The survey consisted of a single ratings scale item: Rate your experience using a mobile device to connect to a social worker (1 = worst possible experience; 5 = best possible experience), and two qualitative open response questions; (1) What did you like best about this way of connecting to a social worker? (2) What did you like least about this way of connecting to a social worker? The survey also asked if the patient would accept a brief phone call within the week to share more of their opinions about the virtual social work visit. The brief survey was created to require only a few minutes for completion. The study assistant collecting survey data from patients also conducted the brief semi-structured qualitative interview over the phone to those who agreed to receiving a call. Within these brief, informal conversations, the study assistant asked patients what they remembered about the virtual visit, what they liked best and liked least, their expectations for the visit, any resources or follow-up they received, their familiarity and comfort with mobile/tablet technology, and whether they would recommend virtual visits to patients at clinics without on-site social workers. These brief interviews were structured as informal conversations and were not recorded. Rather, the study assistant, with years of experience with qualitative interviewing and data analysis, took brief notes including verbatim quotes from participants.

#### Social Worker Data

Our study social worker (BB) was an experienced primary care social worker, with many years of experience with face-to-face and telephonic patient visits. She was new to virtual visit technology and format. BB was from another clinic, so was unfamiliar with the providers participating in this pilot study, to better replicate experience of virtual visits with off-site social work not embedded in the clinic. BB kept a spreadsheet of patient and virtual visit information to track visit information such as reason for referral, referring provider, number of minutes connected, technology comments, and clinical recommendations/results of the consultation (e.g., referral, resources, services). The study assistant also interviewed BB about her experience delivering virtual visits, assessing what she liked best, least, feedback about the technology, and her opinion about patient experience based on her observations.

#### Primary Care Provider Data

Providers assisting with this pilot study agreed to participate in a brief interview. The study assistant arranged these interviews by phone or in person at the provider's convenience to minimize disruption to clinical practice. These brief informal conversations included questions about the pros and cons of virtual social work visits using mobile device, the likelihood they would use this approach with patients, and how often they think they would use this approach to social work consultation.

#### Approach to Analysis

Qualitative data from patient surveys, interviews, and provider surveys were coded by qualitative researchers not involved in delivery of the virtual visits (JH and KV). Data was coded independently using methods of content analysis to organize feedback into themes (i.e., similar perceptions, experiences, or recommendations described by multiple participants).

## Results

During the initial trial at the first site, primary care providers initiated the process of virtual social work visits with tablet during the medical encounter for 18 patients. Of those referred, 15 resulted in a virtual visit. For clinical and logistical reasons, two patients were instead contacted by telephone and an alternate plan was made for a third patient. Ten of the 18 patients completing a virtual visit completed the brief survey immediately following the visit, and of those 10, 7 agreed to be contacted for a phone follow-up to collect more feedback. Of the 13 providers who made referrals for virtual consultations, 7 were available and completed a brief interview.

Our results included data from 22 social work visits over a 37-day timeframe. Two family practice sites were used. Roughly 82% of the visits used a tablet allowing a video conference and 3 encounters were conducted over the phone. The majority (68%) of the encounters involved adult patients (>18 years of age) with the average age being 33 (range 13-94). Over half (55%) of our patients triaged were male. The average time for the patient-social worker encounter was 44 min (ranging from 15 to 120 min). The SW staff allowed the length of the calls to flex with the subject matter and time available to see the patient. The majority of requests for utilization of the Social Work services were related to questions regarding community resources (45%) and mental health triage (45%). Of the 22 visits, no cases were sent to the emergency department, despite 3 cases being referred for a crisis/emergency assessment (previously these clinics would have sent these patients to an Emergency Department for this assessment).

Ten patients completed a brief satisfaction survey after using the tablet. Their feedback was based on a Likert scale from 1 to 5 (1 being worst and 5 being best). The mean rating of experience ranked 4.7/5 (*SD* = 0.49) with a score range from 4 to 5. All who participated in the survey indicated that they would recommend this service to other patients. Six of these patients agreed to be called for a brief interview regarding their experience. Qualitative themes from patient interviews included convenience (e.g., 004, female: *It's right there. No waiting for appointment…great way to take advantage of technology*.), technology challenges (e.g., 002, male, *There was a slight lag in the video and the sound;* 003 male, *I'm not an IT guy, so when the session has ended make sure it actually logs off*; 006 female: *the nurse setting it up had trouble getting the picture right and trouble with the cord too*), recommendation of tele social work for other patients (e.g., 007 male, *I would recommend it, I think it's a good idea actually*; 004 female *Absolutely I do* [recommend it]).

Seven primary care providers provided feedback after using this resource. Their feedback included themes of convenient, timely access to care (006, clinician at South clinic location: *I truly do not think the patient would have come in to see a social worker otherwise. As a provider, I cannot tell you how much better I felt knowing he had a visit and a plan was made when the patient needed it most)*, and potential time savings for clinicians (e.g., 007, clinician at North clinic location, *I kept moving doing other stuff—kept working while the patient and social worker talked. Time-wise that was very useful)*.

Feedback was also obtained from the social worker (BB) involved in this pilot study, with positive reviews overall. She noted particular benefit from the timely nature of the access to patient care, and the ability to see the faces of patents and staff. In addition, she indicated that patients of all ages appeared comfortable utilizing the technology and were gracious with trying new things and adapting to new situations. The social worker also noted that the concept of using a tablet (rather than an in-person encounter) was not a barrier with rapport. However, technical difficulties were perceived as a potential barrier to communication and care delivery. She provided these examples of technology challenges: Example 1: *I was doing a suicide assessment on an adult individual and there was a delay and an audio break up during an important question. I almost missed a facial expression that was not congruent with the response. I was able to go back and repeat the question, noting the facial response, for a more accurate account. Had I missed that, which I probably would not have in person, there could have been a very different outcome*. Example 2: *I was doing a trauma assessment on a young person and there were a number of break ups in the audio, as well as, difficulty hearing them speak. They were crying and speaking softly, because what they were talking about was difficult for them. It is hard to be attentive to the needs of the patient when the audio is breaking up during a serious and difficult discussion*. Example 3: *There was one instance where the family member of a patient had an angry look and I asked if I had missed something and they said the visual and verbal delays made her frustrated. Despite this, almost every patient and family member I spoke with appreciated the immediate response and ability to access services in a quick and efficient manner*.

## Discussion

Considering that up to 80% of chronic illness and 90% of mental health problems are managed in the primary care setting (14), the need for evidence on practical interventions which can improve access to appropriate resources for social and psychosocial health, and thereby, health outcomes is paramount. This is of importance considering the time and resources are not always available for the individual primary care physician to address each individual social determinant of health (3, 4). Implementation of a social worker via tele communication allows a trained professional to be available to immediately link in to mitigate social issues, prepare the patient for therapy, and/or assist with alternative options to higher cost of care (such as emergency department visits).

Within the limitations of this in-practice pilot study it was demonstrated that social work triage was possible and valued by patients and providers for adults and children in busy primary care practices when delivered via a tablet, despite technical challenges. Patients of all ages seemed to accept the use of a virtual social worker without great difficulty. The PCPs were easily able to include this virtual resource into their busy workflow. The main challenges described in our feedback centered around encounters where the tool itself was not working well (not the concept of using a virtual tool for evaluation). It is worthy to note that in the timeframe of data collection, analysis, and manuscript authorship, the technological advances –catalyzed by the coronavirus pandemic—have greatly improved the quality of the technology available to implement these virtual encounters. Thus, as technology continues to improve, the major drawbacks of technical and connection interruptions described in our study will likely continue to improve.

Considering that technology and connection interruptions were the major focus of negative feedback, practical methods to counteract this dilemma would be appropriate. For instance, one lesson learned for practical implementation could include having a backup tablet available, or utilization of other modes of communication (such as a phone call) to properly finish the encounter if interrupted. Furthermore, having staff trained in basic tablet/connection troubleshooting can be quite beneficial in these predicaments. These connection interruptions in the context of emergency situations, such as suicide risk assessments, are notable, and further implementation strategies can be considered that are similar to the best practices when conducting risk assessment interviews in person. This would include for virtual visits where the topic is already known to include suicidality, having staff, such as nursing staff, sit with the patient when alone, and during the video encounter to ensure patient's safety if technology and/or connection interruptions occur. As it is best practice to have a policy of approach for suicide risk assessment and action when a patient is voicing suicidal thought in person, similar policies would be appropriate in the context of the virtual clinical environment.

With the model of virtual use of a tablet, a centralized group of social workers could provide services for a number of clinics in a geographic area where they know the resources, thus limiting the need for small clinics to hire a portion of a social worker and the cost of transportation of a social worker driving to these sites. Furthermore, considering there are already models of social work staff providing virtual services to emergency rooms, these same social workers would make an excellent resource to create a pool of SW covering PCP practices. This is of value as these SW could use their experience for triaging patients to better help differentiate management of patients in distress between interventions in the primary care setting vs. presenting to the emergency department. The potential benefit of preventing unnecessary emergency room or hospital services may be even higher in rural areas considering the lack of social work resources (6). With excellent patient and provider satisfaction ratings, this project may provide pilot data for a larger and more rigorous study of clinical and cost outcomes.

## Limitations

This project includes the standard limitations seen in pilot studies: patients were not randomly assigned, outcome measures were limited to satisfaction surveys/interview, and there was no comparison group. Furthermore, conclusions are limited to the setting where the study was tested, thus cannot be expected to fully extrapolate to clinics in different locations and populations. Both sites involved in the project had experience with on-site social work services which may bias their acceptance of this option. The project focused on the initial contact between a social worker and a new patient, and not on follow up visits and psychotherapy which are other roles of social workers in primary care sites.

## Conclusion

A geographically centralized social work service linked to several primary care clinics via a telemedicine resource is an attractive option to allow a social worker to virtually “step into” the office (using telemedicine) of a primary care physician and take over the care of a patient enough to identify and address social determinants, options for behavioral health, and potentially ways to avoid higher cost emergency services.

## Data Availability Statement

The raw data supporting the conclusions of this article will be made available by the authors, without undue reservation.

## Ethics Statement

The studies involving human participants were reviewed and approved by Mayo Clinic Institutional Review Board. Written informed consent to participate in this study was provided by the participants' legal guardian/next of kin.

## Author Contributions

KV and MW contributed to conception and design of the study. BB organized data from patient encounters. KV performed the statistical analysis. DF conducted the literature search, created the figure, and wrote the first draft of the manuscript. KV, BB, and MW edited sections. DF incorporated edits and expanded content. All authors contributed to manuscript revision, read, and approved the submitted version.

## Funding

The studies involving human participants was presented to the Mayo Clinic Institutional Review Board (IRB). No formal IRB review was required. Only those patients and providers who verbally agreed to be surveyed or interviewed were included and all information was deidentified.

## Author Disclaimer

The contents provided are solely the responsibility of the authors and do not necessarily represent the official views of HHS or any of its agencies.

## Conflict of Interest

The authors declare that the research was conducted in the absence of any commercial or financial relationships that could be construed as a potential conflict of interest.

## Publisher's Note

All claims expressed in this article are solely those of the authors and do not necessarily represent those of their affiliated organizations, or those of the publisher, the editors and the reviewers. Any product that may be evaluated in this article, or claim that may be made by its manufacturer, is not guaranteed or endorsed by the publisher.
